# A Rare Case of Cervico-Mediastinal Thyroid Teratoma

**DOI:** 10.7759/cureus.59560

**Published:** 2024-05-03

**Authors:** Adriana Nocera, Qianqian Zhang, Antonio Giulio Napolitano, Dania Nachira, Elisa Meacci

**Affiliations:** 1 Thoracic Surgery, Fondazione Policlinico Universitario Agostino Gemelli IRCCS – Università Cattolica del Sacro Cuore, Roma, ITA; 2 Pathology, Fondazione Policlinico Universitario Agostino Gemelli IRCCS – Università Cattolica del Sacro Cuore, Roma, ITA

**Keywords:** cervico-mediastinal thyroid teratoma, germ cell tumors, thyroid, mediastinum, teratoma

## Abstract

Teratomas are rare germ cell tumors derived from multiple germinal cell layers. Thyroid teratomas, specifically, are exceptionally uncommon and present unique diagnostic and therapeutic challenges. Here, we report a case of cervico-mediastinal thyroid teratoma, highlighting diagnostic difficulties and surgical management. A 37-year-old woman presented with right lateral cervical swelling, leading to radiological imaging suggesting a thymic teratoma. However, cytology indicated a colloid cyst. Surgical removal was performed, revealing a mixed-type teratoma originating from the thyroid gland. Thyroid teratomas pose diagnostic and therapeutic challenges due to their rarity and complex nature. Further research is needed to establish standardized guidelines for their management.

## Introduction

Teratomas are a widespread form of germ cell tumors and histologically are defined as tissues derived from the 3 germinal cell layers: ectoderm, mesoderm, and endoderm. These cells are capable of differentiating, giving rise to all the various tissues of the organism [1]. Therefore, the conformation can differentiate in several types of tissues: thyroid, bone, muscle, cartilaginous, and tooth buds. Tumors in the head and neck represent 5% of all benign and malignant germ cell tumors and 6% of all teratomas. [2] Teratomas that arise in the neck are rare, and even fewer are located within thyroid tissue [3]. This article highlights the difficulties of a correct diagnostic procedure and correct operative timing in the teratomas of the cervical-mediastinal district, which are extremely rare.

## Case presentation

A 37-year-old woman with no comorbidities and negative medical history, except for a right hemithyroidectomy 14 years before for benign disease, came to our observation for right lateral cervical swelling for some months. Therefore, the patient underwent an MRI describing an oval formation affecting the upper thoracic layer (51x35x66 mm) with non-homogeneous contrast impregnation due to the presence of gross calcification and an area with an adipose component. The formation was contiguous to the thymic tissue and was expressed in the right lateral-cervical compartment until it reached the thyroid plane. A neck-chest CT scan with contrast enhancement was performed, which highlighted an expansive lesion in the anterosuperior mediastinum, with maximum dimensions on the axial plane equal to 62x42 mm and a maximum extension on the coronal plane equal to 85mm.

**Figure 1:**
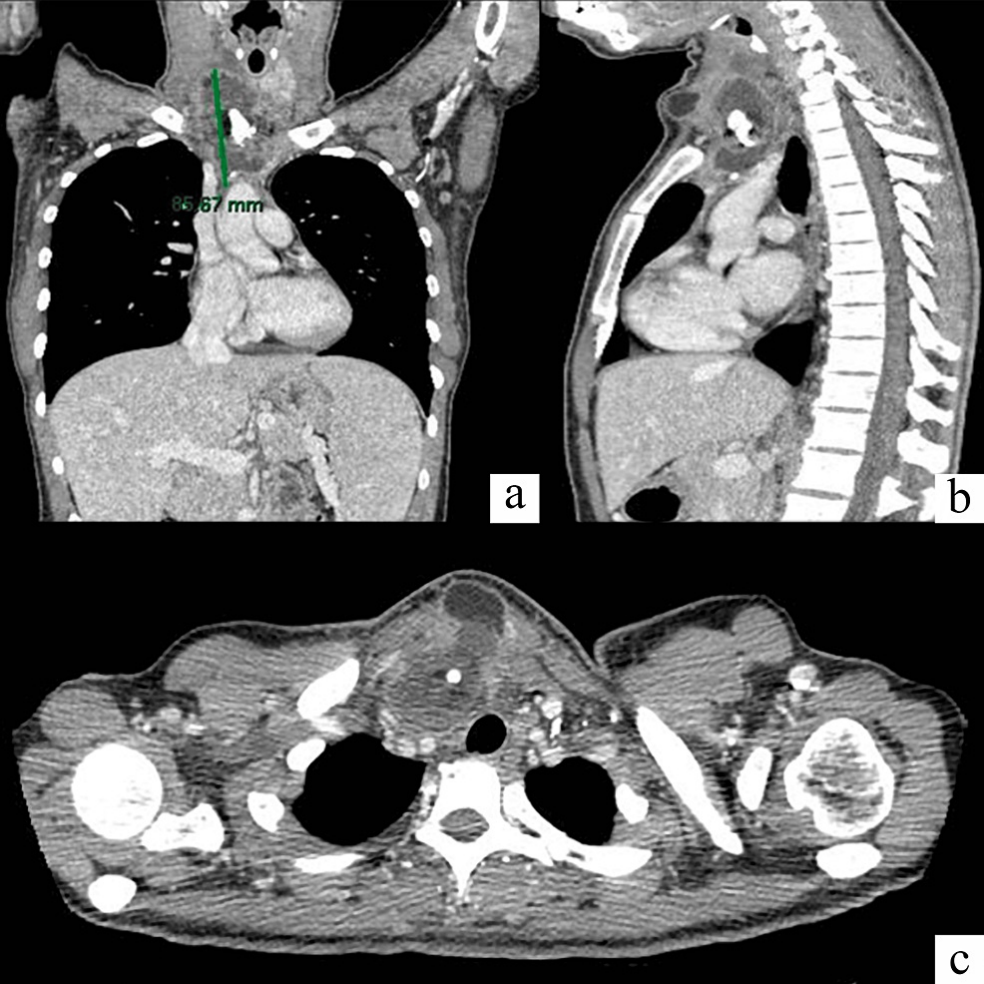
Neck-chest CT scan a) Coronal; b) Sagittal; c) Axial CT scan

This lesion appeared to be in continuity with the thymic tissue, caudally, with the left innominate vein compressed and markedly thinned, posteriorly, and the right innominate vein, the brachiocephalic arterial trunk, and the trachea slightly displaced to the left side. Cranially, it extended into the right thyroid lobe, previously removed. Furthermore, the appearance of a fluid/adipose component density was highlighted, extending anteriorly at the base of the neck in the median area where it reached the skin (Figure 1). Radiological investigations indicated a thymic teratoma.

**Figure 2:**
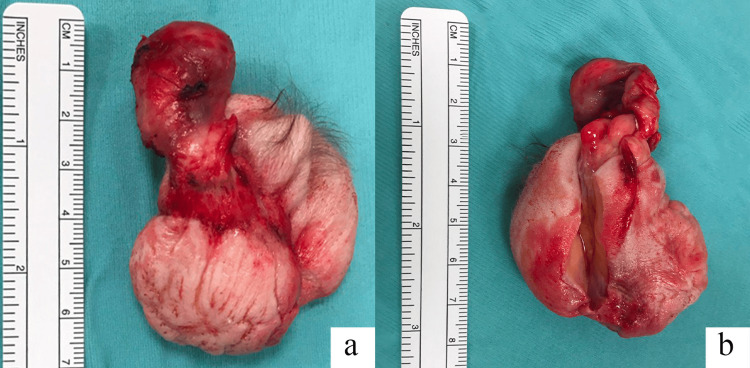
Surgical specimen after removal a) Anterior macroscopic view of the operative specimen; b) Posterior macroscopic view of the operative specimen

Therefore, the patient performed fine needle aspiration of the lesion, which highlighted blood fibrin material, some colloid globules, and very rare cellular elements (TIR1 according to the Italian classification SIAPEC-AIT 2014; Bethesda 1 according to Bethesda system, 2023). Due to the non-diagnostic cytological findings, after a multidisciplinary agreement, surgery was decided to remove the lesion. After fine needle aspiration, the patient developed a chronic inflammatory process with cutaneous fistulisation of the neoformation. Antibiotic therapy was undertaken before surgery. Surgery was performed through a collar cervicotomy. Detachment of the subcutaneous layers and divarication of the sternohyoid muscles and sternothyroid muscles appeared difficult due to tenacious adhesions resulting from a chronic inflammatory process and recent cutaneous fistulization affecting the right thyroid lodge with the skin, with consequent subversion of the vascular-muscular structures. As for teratoma, the neoformation presented a capsule containing calcified nodules, caseous material, and hairs. Surgical removal was performed en bloc after sectioning the peduncle at the thyroid isthmus, and the specimen was sent for definitive histological examination (Figure 2).

**Figure 3:**
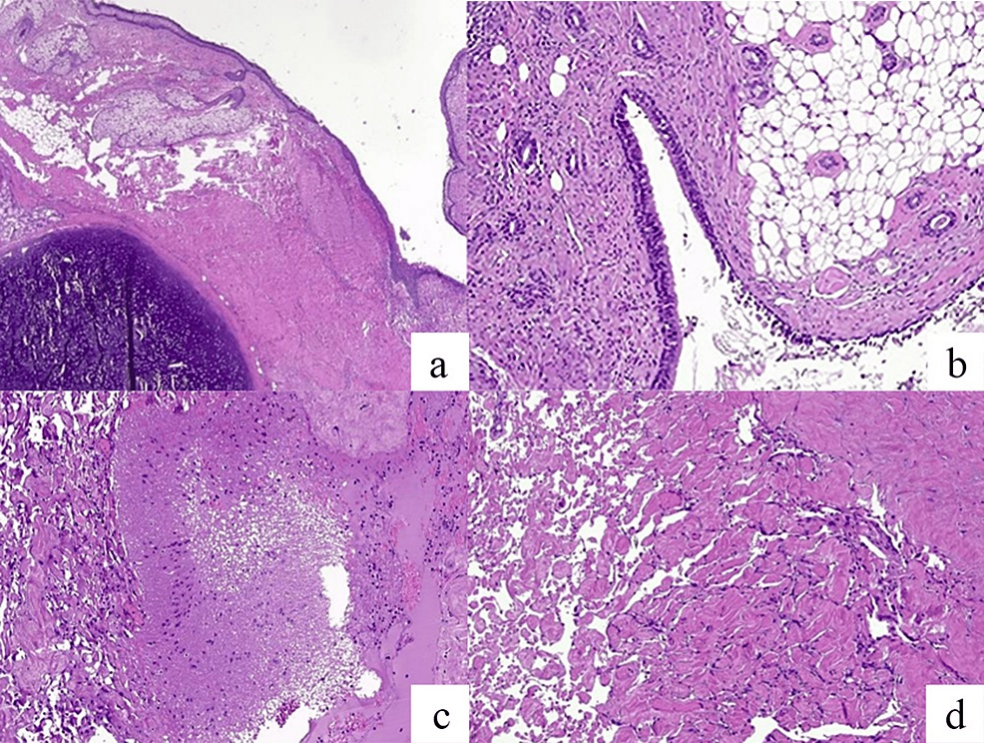
Histological examination a) Hair follicles and sebaceous glands are attached to stratified pavement epithelium. A fragment of cartilage with a small group of bronchial glands was observed; b) Fibrous cystic wall containing adipose tissue and lined by respiratory epithelium, focally pseudostratified; c) mature nervous tissue; d) the stroma shows focal pseudopapillary changes, resembling a Masson phenomenon

The left thyroid lobe showed no signs of disruption and was left in place. An accurate skin surgical closure included the previous fistulous passage between the lesion and the skin. On microscopic examination, skin and cutaneous appendage, commonly found in mature teratoma, were seen (Figure 3a). Respiratory mucosa, adipose tissue (Figure 3b), and mature brain tissue (Figure 3c) were clearly identified. In this case, an interesting pseudopapillary stroma change was observed, resembling morphologically the intravascular papillary endothelial hyperplasia (Figure 3d).

After an accurate examination, no immature component was found. Based on these histologic and macroscopic findings, mature teratoma was diagnosed. After four months after surgery, the patient performed a new contrast CT scan that did not document signs of macroscopic disease recurrence. The patient did not undergo any post-surgery therapy and was alive and disease-free at the last follow-up of 16 months.

## Discussion

Various theories propose the origin of teratomas, one of which involves migrating primordial germ cells from the extraembryonic mesoderm to the genital ridges. Others suggest that during the fourth and fifth weeks of gestation, among the endodermal cells of the yolk sac that migrate to the gonadal ridges, some may not reach their intended destination and cause teratomas to develop anywhere between the brain and the coccygeal area, typically in the midline [1].

Mature teratomas are generally benign tumors whose structure is characterized by more than one mature differentiated tissue, such as skin, hair, teeth, muscle, and nervous tissue. They grow slowly and are almost always asymptomatic, so much so that they are occasionally discovered during radiodiagnostic tests performed for other reasons. They can rarely cause symptoms when they grow and compress vital structures, causing dyspnoea [2]. Primary thyroid teratomas are rare thyroid gland neoplasms of germ-cell derivation that display features of trilineage differentiation [3]. Benign lesions of thyroid teratomas are more commonly observed in children or infants, whereas malignant cases are predominant among adults [4]. Malignant thyroid teratomas typically manifest with an initial neck mass, often accompanied by lymphadenopathy, which can progress to metastasis, primarily affecting the lungs [3].

The thyroid gland is accepted as the site of origin for the teratoma if (i) the tumor occupies a portion of the thyroid gland, (ii) there is direct continuity between the tumor and the thyroid gland, or (iii) the tumor has replaced the gland (no thyroid gland is identified intraoperative or by imaging) [5]. Lurje's description of malignant teratoma of the thyroid in adults dates back to 1908 when he reported a case involving a 53-year-old woman. She underwent a hemithyroidectomy, but she died during the surgical procedure [3-4].

Thyroid teratoma diagnosis poses challenges with fine-needle aspiration cytology (FNAC) due to frequently yielding indeterminate or unreliable results [6]. Complications such as teratoma rupture are relatively rare, according to a recent study by Ota E et al. [7], with only 56 cases of mediastinal teratoma rupture reported to date. In the case described by the authors [7], the rupture of a mature teratoma in a 29-year-old girl caused mediastinitis, requiring emergency surgical resection within 24 hours.

As thyroid teratoma is rare, treatment lacks established guidelines. However, most documented cases involve complete surgical excision followed by adjuvant therapy comprising chemotherapy, radiation, or a combination of both. There are a few cases in the literature that underwent just a partial thyroidectomy with chemotherapy and/or radiotherapy and did not require a completion thyroidectomy or complete nodal clearance [3].

In our case, the patient noticed cervical swelling, for which she decided to undertake further diagnostic investigations. The diagnosis was not immediate, and there was a discrepancy between the cytological and radiological examinations without a real guideline for diagnosing teratomas of the cervico-mediastinal district. Furthermore, the lack of adequate studies makes establishing the correct operative timing impossible. In our case, macroscopically, the teratoma had a direct continuity with the thyroid gland [4].

When a tumor comprises immature and malignant components alongside areas resembling mesenchymal and neural tissue, it necessitates a thorough differential diagnosis. This includes distinguishing it from other malignancies such as anaplastic carcinoma, carcinosarcoma, Ewing sarcoma, primitive neuroectodermal tumor, small cell carcinoma, and malignant lymphoma [8]. Also, in our case, especially for the radiological aspects, a tumor with a thymic origin was hypothesized.

The histology of the tumor is indicative of the disease's aggressiveness. Tumors graded as 0 consist solely of mature elements and are generally deemed benign. Those graded as 1 or 2 are considered immature, while grade 3 tumors, characterized by immaturity, are typically classified as malignant. The presence of embryonal carcinoma or yolk sac tumor places teratomas in the malignant category [9]. Diagnosing primary thyroid teratoma presents a challenge to pathologists due to its complexity and rarity.

According to a recent scientometric analysis on teratomas conducted by Murat Çalbiyik and Sinan Zehir, Italy is the tenth country (136 publications, 3.2%) that contributed to the global literature on teratomas from 1980 to 2022. The analysis revealed that the topics most studied are ovarian teratoma, ovarian dermoid cysts, sacrococcygeal teratoma, and immature teratoma types. After these teratomas, they focus on topics related to mediastinal teratoma, testicular teratoma, and retroperitoneal teratoma [10].

## Conclusions

There are no standard strategies for treating thyroid teratoma that can help clinicians make decisions for each case. Correct surgical timing is essential to avoid complications. Our study aimed to highlight the need for further studies on this pathology.
